# Binding Affinity and Mechanisms of Potential Antidepressants Targeting Human NMDA Receptors

**DOI:** 10.3390/molecules28114346

**Published:** 2023-05-25

**Authors:** Simin Ye, Yanqiang Han, Zhiyun Wei, Jinjin Li

**Affiliations:** 1 Shanghai Key Laboratory of Maternal Fetal Medicine, Shanghai Institute of Maternal-Fetal Medicine and Gynecologic Oncology, Shanghai First Maternity and Infant Hospital, School of Medicine, Tongji University, Shanghai 200092, China; yesm777@sjtu.edu.cn; 2 Key Laboratory for Thin Film and Microfabrication of Ministry of Education, Department of Micro/Nano-Electronics, Shanghai Jiao Tong University, Shanghai 200240, China; hanyanqiang@sjtu.edu.cn

**Keywords:** depression, NMDA receptor, ketamine, ligand–protein docking, binding free energy, drug design

## Abstract

Depression, a mental disorder that plagues the world, is a burden on many families. There is a great need for new, fast-acting antidepressants to be developed. N-methyl-D-aspartic acid (NMDA) is an ionotropic glutamate receptor that plays an important role in learning and memory processes and its TMD region is considered as a potential target to treat depression. However, due to the unclear binding sites and pathways, the mechanism of drug binding lacks basic explanation, which brings great complexity to the development of new drugs. In this study, we investigated the binding affinity and mechanisms of an FDA-approved antidepressant (S-ketamine) and seven potential antidepressants (R-ketamine, memantine, lanicemine, dextromethorphan, Ro 25-6981, ifenprodil, and traxoprodil) targeting the NMDA receptor by ligand–protein docking and molecular dynamics simulations. The results indicated that Ro 25-6981 has the strongest binding affinity to the TMD region of the NMDA receptor among the eight selected drugs, suggesting its potential effective inhibitory effect. We also calculated the critical binding-site residues at the active site and found that residues Leu124 and Met63 contributed the most to the binding energy by decomposing the free energy contributions on a per-residue basis. We further compared S-ketamine and its chiral molecule, R-ketamine, and found that R-ketamine had a stronger binding capacity to the NMDA receptor. This study provides a computational reference for the treatment of depression targeting NMDA receptors, and the proposed results will provide potential strategies for further antidepressant development and is a useful resource for the future discovery of fast-acting antidepressant candidates.

## 1. Introduction

Major depressive disorder (MDD) is a widespread mental illness that limits people’s quality of life and is expected to rank first in the global burden of disease by 2030, according to the WHO [1]. Unfortunately, the main problem is that our understanding of the etiology and pathophysiology of major depression is incomplete and there is as yet no detailed understanding of its mechanism to guide future treatments. The current mainstream monoamine hypothesis [2,3] cannot fully explain the pathological features of depression, and some patients with depression respond poorly to existing antidepressants.

A growing body of preclinical research has demonstrated that N-methyl-D-aspartic acid (NMDA) glutamate receptors play a role in the pathophysiology of major depression and the mechanism of action of antidepressant therapy. Many NMDA receptor antagonists show rapid clinical antidepressant and antidepressant effects, according to recent studies [4,5]. NMDA receptors can bind with the excitatory neurotransmitter L-type glutamate, which then mediates neuronal development, synaptic transmission, and many other physiological and pathological processes. Dysfunction in NMDA is associated with a variety of neurological disorders, such as schizophrenia, Alzheimer’s disease, bipolar disorder, and post-traumatic stress disorder. The GluN1 and GluN2 subunits of the NMDA receptor consist of an extracellular n-terminal structural domain (NTD), an agonist-binding structural domain (ABD), a transmembrane structural domain containing an ion channel (TMD), and an intracellular C-terminal structural domain (CTD) [6,7]. The TMD region consists of three transmembrane helices (M1, M3 and M4), and a pore ring consisting of the M2 helix and an extended region. The TMD tetramer forms an ion-permeable pore whose gating is controlled by NTDs and ABDs [8]. The extended region forms the narrowest part of the ion channel and is called the selective filter. The structure of the NMDAR selective filter is specifically responsible for ion recognition, identification and permeation, and interaction with multiple pore-blocking drugs [9]. Recently, Zhang et al. [10] studied the cryo-electron microscopic structures of human GluN1-GluN2B NMDA receptors in a complex with S-ketamine, glycine, and glutamate. The binding pocket of S-ketamine was found in the central vestibule of the TMD region. Therefore, the discovery or development of drug molecules that can bind to the TMD region of NMDA and inhibit its function is an effective way to resolve the current hypothesis.

Ketamine, a non-competitive channel blocker of NMDA receptors, is reported to be effective in animal models of depression [11,12]. Ketamine is a racemic mixture consisting of equal amounts of S-ketamine and R-ketamine. Previous studies suggested that R-ketamine might be a safer antidepressant without side effects than (r, s)-ketamine and S-ketamine [13,14]. Like ketamine, memantine is a derivative of amantadine [15], a drug used to treat Parkinson’s disease that has been shown to act as a non-competitive NMDA antagonist with low affinity and rapid kinetics of blocking and unblocking the channel [16]. Ro 25-6981 is a novel and highly selective NR2B antagonist with a higher affinity for NR2B than conventional NR2B antagonists such as ifenprodil [17,18]. We selected eight promising NMDA receptor antagonists based on pioneering work [19,20] (S-ketamine, R-ketamine, memantine, lanicemine, dextromethorphan, Ro 25-6981, ifenprodil, and traxoprodil) and performed high-precision quantum calculations to find the most effective inhibitor. Table 1 shows the chemical structure of the eight antidepressants selected for this study. Of the eight drugs, ifenprodil [21] and traxoprodil are widely used to treat Alzheimer’s disease. For depression, there are not enough clinical trials. Among the ifenprodil site antagonists, we selected Ro 25-6981, which is the (R,R)-isomer of ifenprodil, because it has been reported to have rapid and sustained antidepressant effects in animal models without psychotomimetic side effects or abuse liability. The other isomers of ifenprodil have not been extensively studied for their antidepressant potential or mechanism. These substances can be classified into two groups: dizocilpine-site antagonists (ketamines, memantine, dextromethorphan) and ifenprodil site antagonists (ifenprodil, traxoprodil, Ro 25-6981). Glutamate site antagonists and glycine site antagonists were not included in this study because their antidepressant potential is still unclear or controversial [22,23].

The simulation of molecular dynamics affects a variety of disciplines, including physics, chemistry, biology, and materials, by linking the properties of microscopic systems to those of macroscopic systems using computer calculations. Abrusán et al. used in silico analyses to investigate whether the structural similarity of the binding sites affects the metastable process [24]. Focusing on molecular simulation studies, Feng et al. explored the microscale structure information of short curdlan to reveal its multi-chain conformational behavior [25]. The activities of numerous drugs and other biomolecules are expressed through interactions with receptor macromolecules. Therefore, the binding free energy between receptor and ligands is a central issue in structure-based computational drug design. Based on the structure of a protein and a small molecule ligand, it is possible to simulate various information about the conformational changes of the whole system over time using molecular dynamics (MD) methods and obtain the trajectory of the whole system, which can help to further investigate the motion characteristics of the active site of the protein during the binding of the ligand molecule.

The NMDA receptor is widely involved in brain function and disease, and to find new sites of drug action will help to understand the mechanism of antagonist and NMDAR inhibition and provide more possibilities for subsequent drug development. In this study, we investigated the binding affinity and mechanism of an FDA-approved antidepressant (S-ketamine) and seven potential antidepressants (R-ketamine, memantine, lanicemine, dextromethorphan, Ro 25-6981, ifenprodil, and traxoprodil) targeting the NMDA receptor. The TMD region (GluN1-GluN2B) was selected as the drug target, of which a 3D structure was recently published by the Zhang group [10]. We performed ligand–protein docking and molecular dynamics simulations to study the interactions between the NMDA receptor and drug molecules and evaluated the binding affinity by calculating binding free energies, providing potential theoretical guidance for further drug molecule design.

## 2. Results and Discussion

We performed molecular dynamics simulations using Amber to study the interactions between receptor proteins and drug molecules and evaluated the strength of the interactions by calculating binding energies. We also analyzed the specific residues that contributed significantly to the binding energy, which provided theoretical guidance for further drug molecule design. For each drug, we generated 10,000 structures of the NMDA receptor and drug–ligand complexes based on the Rosetta3 program. Among the 10,000 structures, the scoring mechanism in Rosetta3 first filtered out the structures where the drug was not in contact with the protein. Among the remaining structures, Rosetta3 overall scoring was combined to filter out the most likely 3D conformation.

Table S1 in the Supplementary Materials shows the Rosetta total results, binding energies, and some other energy reference values of the best docking complex for the eight drugs obtained by the docking procedure. The data in the table show that the Rosetta scores of the eight drugs were not much different, with traxoprodil having the lowest total Rosetta score and binding energy among the selected drugs. All eight drugs had docking binding energies below −7.2 kcal/mol and total energy scores below −144 kcal/mol with the NMDA receptor. The Rosetta docking results indicate that the eight selected drugs have fair binding affinity to the NMDA receptors and can be subjected to the next step of computational simulation.

The screened composite structures were used for molecular dynamics simulations to investigate the binding capacity and interaction between the drug and the NMDA receptor. Taking S-ketamine as an example, the ligand–protein complex system became stable with a convergence of temperature, density, and total energy after a 2-ns pre-equilibrium run. Then a production run of 40 ns was performed, and the system remained stable. The stability of the ligand–protein system during the simulation was investigated by root-mean-square deviation (RMSD) analysis.

**Figure 1:**
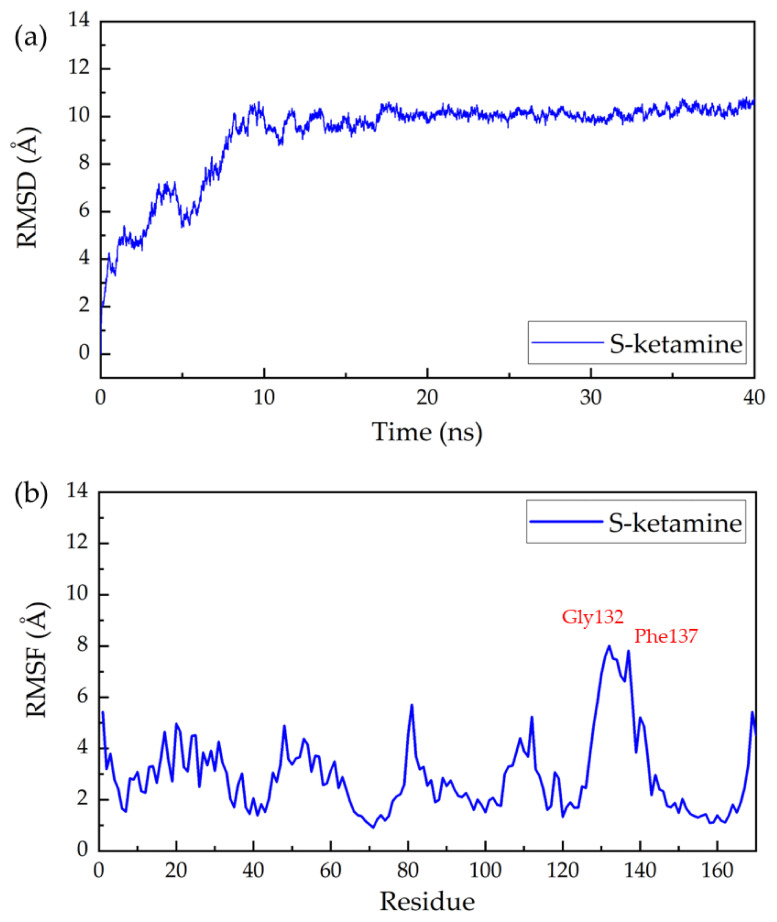
Root mean square deviation (RMSD) and root mean square fluctuation (RMSF) of S-ketamine and NMDA receptor complex in a 40-ns MD simulation: (**a**) the RMSD and (**b**) the RMSF of all non-hydrogen atoms in the NMDA acceptor throughout the simulation.

The RMSD of all non-hydrogen atoms of the complex of NMDA receptor and S-ketamine throughout the production simulation is shown in Figure 1a. The protein backbone of the ligand–protein complexes remained stable after 10 ns simulation. Root mean square fluctuation (RMSF) analysis was also performed for the complex to characterize the degree of freedom of protein residue motion in kinetic simulations. As shown in Figure 1, Gly132, Phe137, Glu131, Ala133, Pro134, Gly130, Arg135, Ser136, Ser138, Ile129, Arg112, and Arg140 showed a high increase and decrease during the simulation. This may indicate that these regions have higher flexibility and are important for the protein to perform its function. The potential significance of these elastic regions is that RMSF-labeled residues may affect structural stability or belong to the active site of the protein. The RMSDs and RMSFs of the complex of the rest of the seven drug molecules can be found in Supplementary Materials (Figures S1, S5, S8, S11, S14, S17 and S20). From these figures, we can see some differences in the positions of the residues that undergo significant positional changes when different drugs interact with the receptor, which may indicate that different drugs tend to bind at different sites.

The binding free energy of eight drugs to the NMDA receptor was calculated using molecular dynamics methods. The total binding free energy (ΔG_bind_) was determined by the generalized binding free energy of molecular mechanics according to the Born surface area (MM-GBSA) and is a common method in the final state approach [26], which aims to calculate the difference in binding free energy of two molecules in solution [27]. The MM-GBSA method can be used to calculate the binding free energy between protein and ligand. The total binding energy is composed of two components, total enthalpy, and total entropy, where the total enthalpy was determined by the MD simulation and the total enthalpy included components of gas-phase energy and solvation energy. The entropy contribution to the binding free energy was calculated by the normal mode (nmode) [28] analysis method. We calculated the binding free energy ΔG_bind_ between the drug and the NMDA receptor using MM-GBSA and nmode. Table 2 describes the binding free energy as well as other energy subcomponents that make up the binding energy, including the gas-phase of free energy (∆E_gas_), solvation enthalpy (∆E_solv_), total enthalpy (ΔE_total_), entropy contribution (TΔS_nmode_), and total binding free energy (ΔG_bind_).

**Figure 2:**
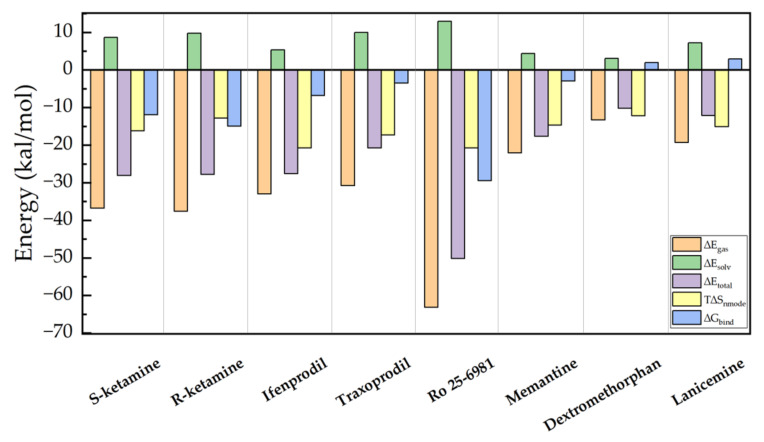
Binding free energies and components of eight drugs with NMDA receptors, including ∆E_gas_, ∆E_solv_, ∆E_total_, T∆S_nmode_ and ∆G_bind_.

To better compare the binding energies of the eight drugs, we plotted the data from Table 2 in Figure 2. As can be seen in the figure, the total enthalpy ∆G_bind_ (blue column) shows that of the eight drugs, Ro 25-6981 has the strongest binding energy to the NMDA receptor, as −50.1926 kcal/mol. The calculation of entropy change also has a significant impact on the calculation of binding free energy, which could be due to the changes in the complex system caused by the binding. After calculating the contribution of entropy value to binding energy, Ro 25-6981 has the lowest binding free energy (−29.4311 kcal/mol), followed by R-ketamine (−14.9605 kcal/mol), S-ketamine (−11.9223 kcal/mol), ifenprodil (−6.8353 kcal/mol), traxoprodil (−3.5063 kcal/mol), and memantine (−2.9621 kcal/mol).

**Figure 3:**
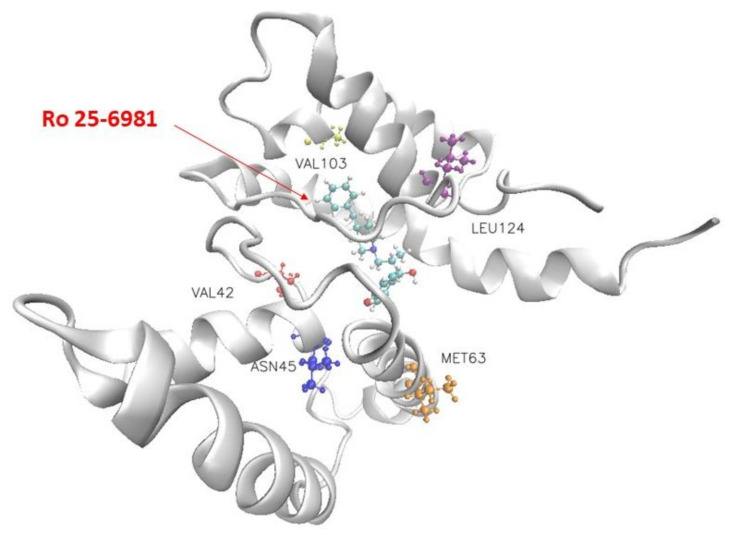
Visualization of the docking conformation of the NMDA receptor and Ro 25-6981. The ball-and-stick model shows the binding pocket of five residues in the NMDA receptor with dominant binding contributions to Ro 25-6981.

Figure 3 shows the structure of Ro 25-6981 and the NMDA receptor and the five residues that contribute most to the binding interaction. The blue, yellow-orange, red, and pink molecules in Figure 3 indicate Leu124, Met63, Asn45, Val103, and Val42, respectively. Table 3 lists the top five residues that contribute substantially to the binding of the eight selected drugs to the NMDA receptor. Our molecular docking results showed that Ro 25-6981 had the strongest binding affinity to the NMDA receptor among the eight selected antidepressants, with a binding free energy of −29.431 kcal/mol. This is consistent with a previous study by Fischer et al. [17], who reported that Ro 25-6981 is a potent and selective antagonist of NMDA receptors containing the GluN2B subunit, with an IC50 value of 0.009 μM for GluN2B vs. 52 μM for GluN2A subunits. To further investigate the binding mechanism of Ro 25-6981 and other antidepressants with the NMDA receptor, we analyzed their interactions with key residues in the GluN2B subunit using Amber MMGBSA. As shown in Figure 3, Ro 25-6981 formed four hydrogen bonds and six hydrophobic interactions with Leu124, Met63, Asn45, Val103, Val42 and other residues in the GluN2B subunit. In contrast, other antidepressants formed fewer or weaker interactions with these residues. These results suggest that Ro 25-6981 has a high affinity and specificity for the GluN2B subunit of the NMDA receptor, which may contribute to its antidepressant effects.

**Figure 4:**
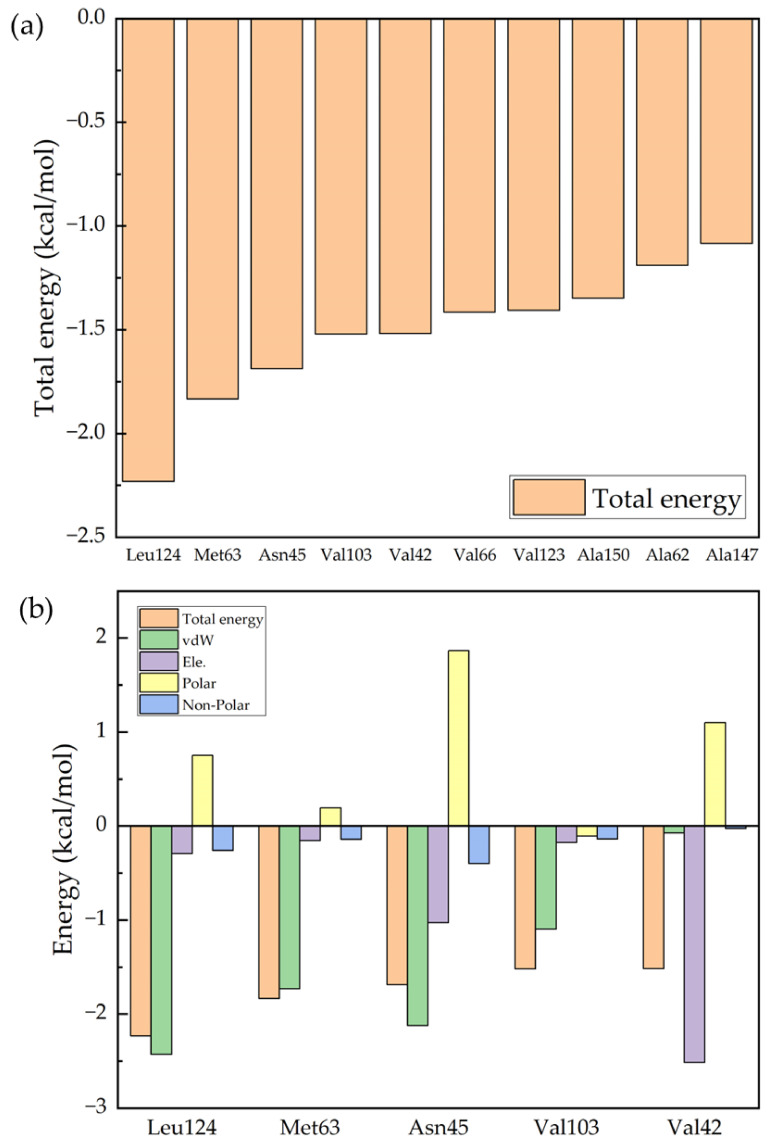
Decomposition of the binding free energy of key residues: (**a**) the 10 key residues with dominant binding contributions for Ro 25-6981 to the NMDA receptor; and (**b**) decomposition of the energy of the five key residues to Ro 25-6981 pairs into four energy terms, namely van der Waals interaction (vdW), electrostatic interaction (Ele.), polar solvation energy (Polar), and nonpolar energy (Nonpolar).

Figure 4a shows the 10 protein residues that contribute most to the binding process of Ro 25-6981 to the NMDA receptor, and Figure 4b shows the analysis of the binding free energy components of the top 5 contributing residues. The figure shows the values of the five energy terms of the residues, namely van der Waals energy (vdW), electrostatic energy (Ele.), polar solvation energy (Polar), and nonpolar solvation energy (Nonpolar). From the figure, we can see that the five residues that contribute the most to the binding process are Leu124, Met63, Asn45, Val103, and Val42. If we further break down the energy of each residue, we can see from Figure 4b that for most of the residues, the van der Waals interaction (green column) plays the most important role. However, for the energy contributed by Val42, electrostatic energy (purple column) plays an important role. From Table S2 in the Supplementary Materials, we can see that this is due to the hydrogen bonding interaction between Val42 and the drug molecule.

S-ketamine is an FDA-approved antidepressant, and R-ketamine is its chiral molecule. In recent studies [29,30,31], R-ketamine was shown to have a stronger potency and a longer antidepressant effect than S-ketamine. R-ketamine is a promising candidate for the treatment of depression because of the lower potential for psychotropic side effects and abuse after ingestion of R-ketamine [32].Therefore, we are interested in comparing the receptor binding energy of S-ketamine with that of R-ketamine. The same conclusion was drawn in our experiments, in which the results of the molecular dynamics simulations showed that the binding free energy of R-ketamine in the TMD region of the NMDA receptor was higher than that of S-ketamine. This might suggest the stronger effect of R-ketamine than S-ketamine. Importantly, compared with S-ketamine, R-ketamine appears to be an effective, long-lasting, and safer antidepressant because of the lower potential for psychotropic side effects and abuse after ingestion of R-ketamine. In addition, we can see in Figure 2 that the two selected drugs, ifenprodil, and traxoprodil, have favorable free energies for binding to NMDA receptors, suggesting that these two drugs may also be considered as antidepressants in the future. Although all eight drugs showed negative binding energies upon molecular docking, both dextromethorphan and lanicemine showed positive binding energies after the molecular dynamics simulations, indicating a low probability of protein–drug molecule binding. This could be due to the drugs not binding to NMDA receptors in the TMD region.

**Figure 5:**
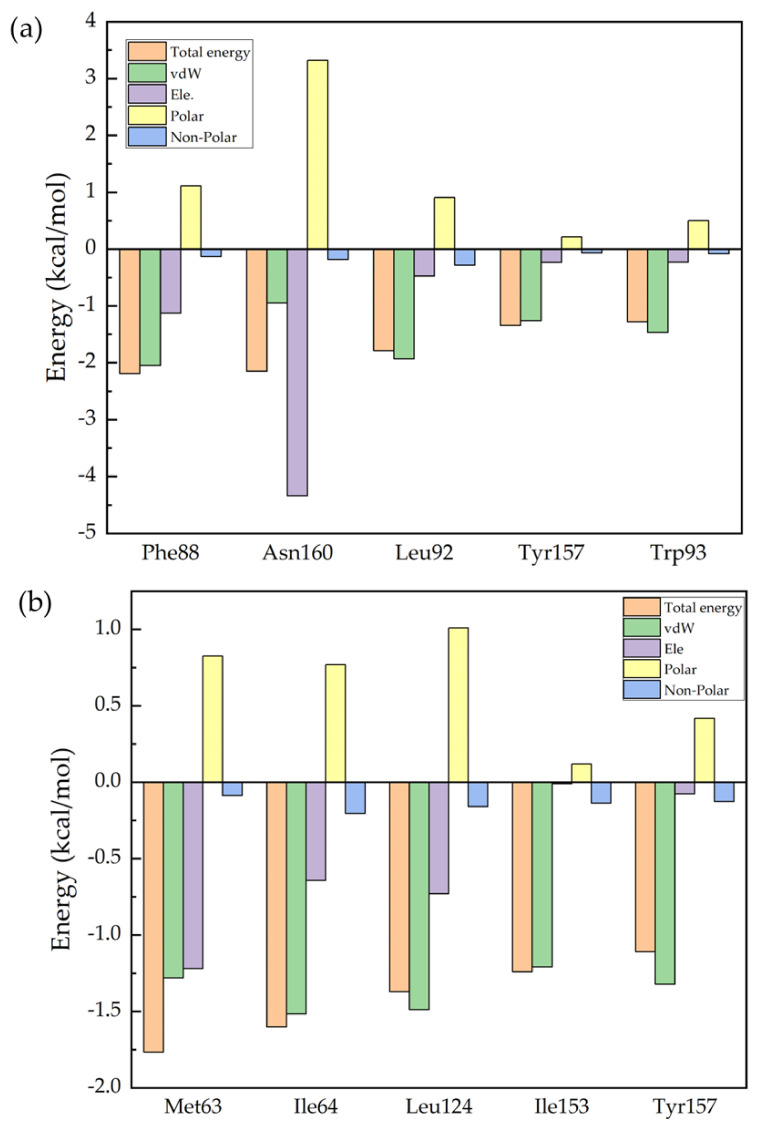
Comparison of the energies of the first five residues which contribute in a predominant way to the binding of S-ketamine and R-ketamine to the NMDA receptor. (**a**) decomposition of the energy of the five key residues to S-ketamine. (**b**) decomposition of the energy of the five key residues to R-ketamine.

Figure 5a,b compares the energies of the top five residues that make dominant contributions to the binding of S-ketamine and R-ketamine to the NMDA receptor. The binding of S-ketamine to the NMDA receptor is mainly due to five residues: Phe88, Asn160, Leu92, Tyr157, and Trp93. The binding of R-ketamine to the NMDA receptor is mainly attributed to Met63, Ile64, Leu124, Ile153, and Tyr157. We see that for these two drugs, although they are mirror isomers of each other, the protein residues that play an important role in the binding to each of the two drugs are not the same. To explore the specific contribution of the protein residues in the binding process to enable better drug design, we further analyzed the specific contribution of each residue of the NMDA receptor. Further decomposing the energy of each residue, we can see in Figure 4b that for both drugs, the dominant role in the energy contribution of residues is still the van der Waals interaction (green column). The difference, however, is that the electrostatic energy (purple column) plays a major role in the energy contributed by Asn160. Table S2 in the Supplementary Materials presents the hydrogen bonding in the eight drugs, and it can also be seen that the nitrogen and hydrogen atoms in Asn160 form hydrogen bonds with the oxygen atoms in S-ketamine, but at the same time, the polar term energy in the solvation free energy is high, resulting in an average value of the overall residue energy contribution. For R-ketamine, it can be seen from Figure 5b that the distribution of its energy contribution per residue is basically the same, and that all of them are higher than those of S-ketamine. From the calculated data, we can assume that R-ketamine has a stronger binding capacity than S-ketamine at this binding site.

The key residues of eight drugs (S-ketamine, R-ketamine, memantine, lanicemine, dextromethorphan, Ro 25-6981, ifenprodil, and traxoprodil) that contribute mainly to the binding free energy and the main energy of the key residues are discussed in the Supplementary Materials (Tables S3–S9, and Figures S3, S6, S9, S12, S15, S18 and S21 ). These may provide some guidance for the design of future antidepressants with a greater affinity for NMDA receptors.

## 3. Method

### 3.1. Protein Structures

The model of GluN1–GluN2B receptor was obtained from the RCSB Protein Data Bank [33], which was determined by Zhang et al. [10] in complex with S-ketamine, glycine, and glutamate. We isolated the TMD region as an individual lobe in the structure as protein receptors. SWISS-MODEL [34], an automated protein structure homology-modeling server, was used to add the missing residues from D582 to L603 in GluN1 and E618 to S626 in GluN2B.

### 3.2. Compounds Selection

Based on previous studies, we selected eight known NMDA receptor antagonists. Six of them (S-ketamine, R-ketamine, memantine, lanicemine [35], dextromethorphan [36], Ro 25-6981, ifenprodil, and traxoprodil [37]) have some clinical trials targeting MDD, while studies with ifenprodil and traxoprodil on the treatment of depression are still ongoing. Structures of eight selected drug molecules were obtained from the PubChem [38] database. We chose both S-ketamine and R-ketamine, hoping to compare their properties. In this study, we investigated the antidepressant effects of the eight drugs on the NMDA receptor.

### 3.3. Ligand–Protein Docking

Most of the drugs in this study lacked actual ligand–protein binding structures, thus we used Rosetta3 [39] to simulate the docking of NMDA receptor proteins and the small drug molecules. The initial site of the ligand starts at the location of the known binding pocket of ketamine to the protein, which is provided by PDB ID: 7EU8. The *Ligand _dock* portion of Rosetta requires a receptor structure (usually a protein) and a small molecule to complete the docking, and it attempts to find a conformation and relative orientation of the two that minimizes the Rosetta score function. The score functions in Rosetta are weighted sums of energy terms, with some representing physical forces such as electrostatic and van der Waals interactions. We obtained 10,000 conformations after Monte Carlo minimization. By clustering ligand poses, we chose the best model from each low-energy cluster. From the 10,000 structures, the 5% lowest-energy candidates were selected based on the fraction of energy they received from the ligand–protein contact structure. Then, the structures with the lowest binding energy were selected as the target structures based on the binding energy of ligands and proteins again for MD simulation.

### 3.4. Molecular Dynamic Simulation

In our study, we used Amber16 software [40] to perform MD simulations and binding energy calculations of drug molecules with the NMDA receptor. In the simulation, the ff14sb and the general Amber force field were used for protein and the small molecule ligand [41], respectively. We used the TIP3P BOX water model for this simulation with at least 12 Angstroms between the NMDA receptor and the periodic box wall. The system was kept neutral by adding Na^+^ and Cl^−^. We used the *sander* procedure to equilibrate the solvated complex by performing a short-time energy minimization. Then, the solvated ligand–protein complex was gradually heated from 0 to 300 K in the NVT ensemble using Langevin thermostat, 100 ps of heating and 100 ps of density equilibration to weakly constrain the complex. Then, an equilibrium run of 1 ns was performed in the NPT ensemble at a temperature of 300 K and a pressure of 1 atm. Finally, we performed 40-ns production runs in the NPT ensemble for each dissolved complex system with a collection interval of 10 ps and collected 3000 frames for further energy calculations.

### 3.5. Binding Free Energy Calculation

Molecular mechanics generalized Born surface area (MM-GBSA) method [42] was used to calculate the binding free energy of small drug molecules and proteins using the MMPBSA.py script in Amber based on the MD trajectories. The overall goal of the MM-GBSA method [42] is to compare the free energies of two different solvated conformations of the same molecule and to calculate the free energy difference between the two states. The equation for calculating the binding free energy using the MM-GBSA method is shown below.

$$\Delta G_{\mathrm{bind}}\;=\Delta E_{\mathrm{total}}\;-\;T\Delta S_{\mathrm{nmode}}.$$

$$\Delta E_{\mathrm{total}}\;=\Delta E_{\mathrm{gas}}\;+\Delta E_{\mathrm{solv}}\;=\Delta E_{\mathrm{vdW}}\;+\Delta E_{\mathrm{ele}}\;+\Delta G_{P}\;+\Delta G_{\mathrm{np}}\;$$

where ΔE_total_ represents the gas-phase of free energy, ΔE_solv_ represents the solvation-phase of free energy, ΔE_vdW_ describes the van der Waals energy and ΔE_ele_ describes the electrostatic energy. ΔG_P_ and ΔG_np_ are used to account for the polar solvation free energy and the nonpolar solvation free energy in the simulation. ΔE_total_ represents the average interaction energy between the receptor and the ligand. ΔS_nmode_ represents the entropy value obtained by the normal mode analysis method [28], and we would normally expect a negative ∆S value for the complex. This means that the ligand is confined near the binding site when the protein and ligand bind to form the complex and that the ligand movement is restricted when the ligand is bound to the protein.

## 4. Conclusions

In summary, based on molecular dynamics simulations, we tentatively determined that Ro 25-6981 had the strongest binding affinity to the NMDA receptor among the eight selected antidepressants, with the binding free energy of −29.431 kcal/mol. We also analyzed the specific residues that contributed significantly to the binding energy. The five residues that contribute most to the binding process of Ro 25-6981 and NMDA receptors are Leu124, Met63, Asn45, Val103, and Val42. In this paper, we also compared two hotly debated drugs, S-ketamine, and R-ketamine, and concluded that R-ketamine had a stronger binding capacity to the NMDA receptor. The results of this study suggest that Ro 25-6981 and R-ketamine are expected to be good candidates for targeting the NMDA receptor in the treatment of depression and provide potential theoretical guidance for the further design of drug molecular structures with the enhanced binding ability to target proteins.

## Figures and Tables

**Table 1:** Chemical structure of the eight antidepressants selected for this study, (S-ketamine, R-ketamine, memantine, lanicemine, dextromethorphan, Ro 25-6981, ifenprodil, and traxoprodil.

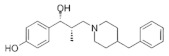	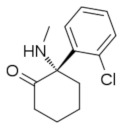	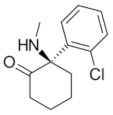	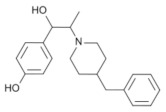
**Ro 25-6981**	**S-ketamine**	**R-ketamine**	**ifenprodil**
C_22_H_29_NO_2_	C_13_H_16_ClNO	C_13_H_17_Cl_2_NO	C_21_H_27_NO_2_
CID: 6604887	CID: 182137	CID: 9838417	CID: 3689
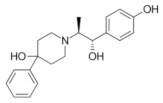	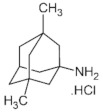	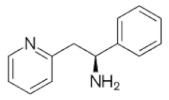	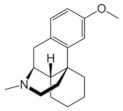
**traxoprodil**	**memantine**	**lanicemine**	**dextromethorphan**
C_20_H_25_NO_3_	C_12_H_21_N	C_13_H_14_N_2_	C_18_H_25_NO
CID: 219101	CID:4054	CID:9794203	CID: 5360696

**Table 2:** Binding free energies of eight drugs with NMDA receptor, where ΔE_gas_, ΔE_solv_, ΔE_total_ TΔS_nmode_, and ΔG_bind_ represent the gas phase enthalpy, solvation enthalpy, total enthalpy, entropy contribution, and total binding free energy, respectively. All energy values are given in kcal/mol.

Conformations	ΔE_gas_	ΔE_solv_	ΔE_total_	TΔS_nmode_	ΔG_bind_
Ro 25-6981	−63.116	12.923	−50.193	−20.762	−29.431
R-ketamine	−37.565	9.795	−27.770	−12.809	−14.961
S-ketamine	−36.777	8.675	−28.102	−16.179	−11.922
ifenprodil	−32.952	5.366	−27.587	−20.751	−6.835
Traxoprodil	−30.773	9.995	−20.777	−17.271	−3.506
Memantine	−22.044	4.409	−17.635	−14.673	−2.962
Dextromethorphan	−13.273	3.056	−10.217	−12.206	1.989
Lanicemine	−19.317	7.180	−12.136	−15.054	2.918

**Table 3:** Top five residues with dominant binding contributions to the NMDA receptor with eight drugs.

Conformations	Important Residues				
	1	2	3	4	5
S-ketamine	Phe88	Asn160	Leu92	Tyr157	Trp93
R-ketamine	Met63	Ile64	Leu124	Ile153	Tyr157
ifenprodil	Leu77	Thr70	Thr158	Leu73	Ala74
traxoprodil	Ile152	Ala155	Leu77	Leu92	Met151
Ro 25-6981	Leu124	Met63	Asn45	Val103	Val42
memantine	Met36	Val15	Trp12	Val19	Trp40
dextromethorphan	Val96	Phe149	Trp146	Arg81	Val154
lanicemine	Ala155	Leu73	Met151	Leu77	Val154

## Data Availability

The data that support the findings of this study are available from the corresponding author upon reasonable request.

## Supplementary Materials

The following supporting information can be downloaded at: https://www.mdpi.com/article/10.3390/molecules28114346/s1. Supporting information provides the visualizations of the docking conformation of the NMDA receptor and eight selected drugs, root mean square deviation (RMSD) and root mean square fluctuation (RMSF) of drug and the NMDA receptor complex, and decomposition of the binding free energy of key residues of eight selected drugs.
