# Supplementary material for: Binding Affinity and Mechanisms of Potential Antidepressants Targeting Human NMDA Receptors
Source: Molecules. 2023 May 25;28(11):4346. doi: 10.3390/molecules28114346 (PMC10254814; doi:10.3390/molecules28114346)
Supplement: Supplementary file 1 [file molecules-28-04346-s001.zip › molecules-2377554-supplementary.pdf]

Supporting Information (SI) for
Binding affinity and mechanisms of potential antidepressants targeting human NMDA receptors

Simin Ye[1,2†], Yanqiang Han[2†], Zhiyun Wei[1*], and Jinjin Li[1,2*]

[1]Shanghai Key Laboratory of Maternal Fetal Medicine, Shanghai Institute of Maternal-Fetal Medicine and Gynecologic Oncology, Shanghai First Maternity and Infant Hospital, School of Medicine, Tongji University, Shanghai 200092, China.
[2]Key Laboratory for Thin Film and Microfabrication of Ministry of Education, Department of Micro/Nano-electronics, Shanghai Jiao Tong University, Shanghai 200240, China.

[†]These two authors contribute equally to this study.
[*]Correspondence to: Jinjin Li (lijinjin@sjtu.edu.cn), Zhiyun Wei (zhiyun_wei@163.com)

Table S1 The total score and important energy terms of the selected eight drugs after docking with Rosetta3. The energy terms include binding energy, Coulombic electrostatic potential, solvation energy, sidechain-backbone hydrogen bond energy (hbond_bb_sc), attractive and repulsive energies between atoms in different residues.

Conformations	Total score	Bind energy	electrostatic	Solvation energy	hbond_bb_sc	attractive	repulsive
**s-ketamine**	-144.654	-9.103	-0.499	288.838	-0.190	-588.516	85.782
**r-ketamine**	-147.176	-8.828	-0.110	289.401	-0.190	-592.509	88.448
**ifenprodil**	-153.57	-12.687	-1.095	289.735	-0.471	-597.576	91.693
**traxoprodil**	-153.915	-13.365	-1.479	290.701	-0.677	-600.377	91.680
**Ro 25-6981**	-152.242	-11.568	0.411	291.098	0.000	-600.546	91.234
**Memantine**	-146.729	-7.231	-0.406	288.884	-0.190	-588.893	86.600
**Dextromethorphan**	-150.362	-10.635	-0.063	289.428	-0.190	-594.154	87.531
**Lanicemine**	-147.129	-8.365	-0.635	289.476	-0.190	-590.556	86.555

Table S2 The hydrogen bond analysis of the selected eight drugs after docking using cpptraj. Acceptor, DonorH, and Donor are the residue and atom name of the atoms involved in the hydrogen bond. AvgDist is the average distance of the bond when present, and AvgAng is the average angle of the bond when present.

Conformations	Acceptor	DonorH	Donor	AvgDist	AvgAng
**S-ketamine**	SKE_170@O1	ASN_160@HD22	ASN_160@ND2	2.8644	158.146
**R-ketamine**	LEU_124@O	RKE_170@H9	RKE_170@N1	2.8816	162.275
	RKE_170@N1	ASN_126@HD21	ASN_126@ND2	2.9043	152.594
**ifenprodil**	LEU_165@O	IF1_170@H27	IF1_170@O2	2.7693	162.539
**traxoprodil**	TRA_170@O2	TYR_69@HH	TYR_69@OH	2.6340	139.755
**Ro 25-6981**	VAL_42@O	RO1_170@H29	RO1_170@O2	2.7397	162.840
	LEU_43@O	RO1_170@H29	RO1_170@O2	2.7294	163.301

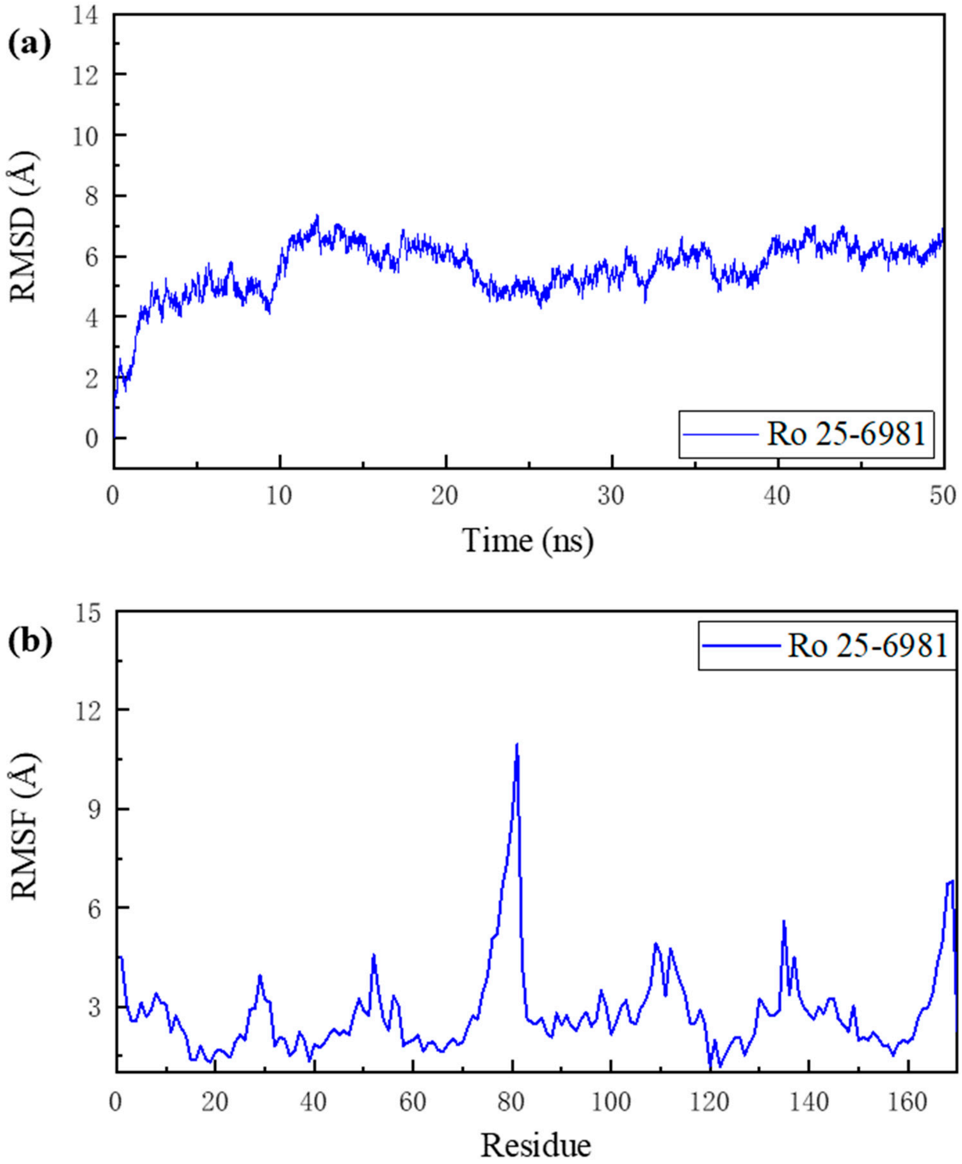

Figure S1 Root mean square deviation (RMSD) and root mean square fluctuation (RMSF) of s-ketamine and the NMDA receptor complex in a 40-ns simulation MD. (a) The RMSD of all non-hydrogen atoms in the complex. (b) The RMSF of all non-hydrogen atoms in the NMDA receptor throughout the simulation.

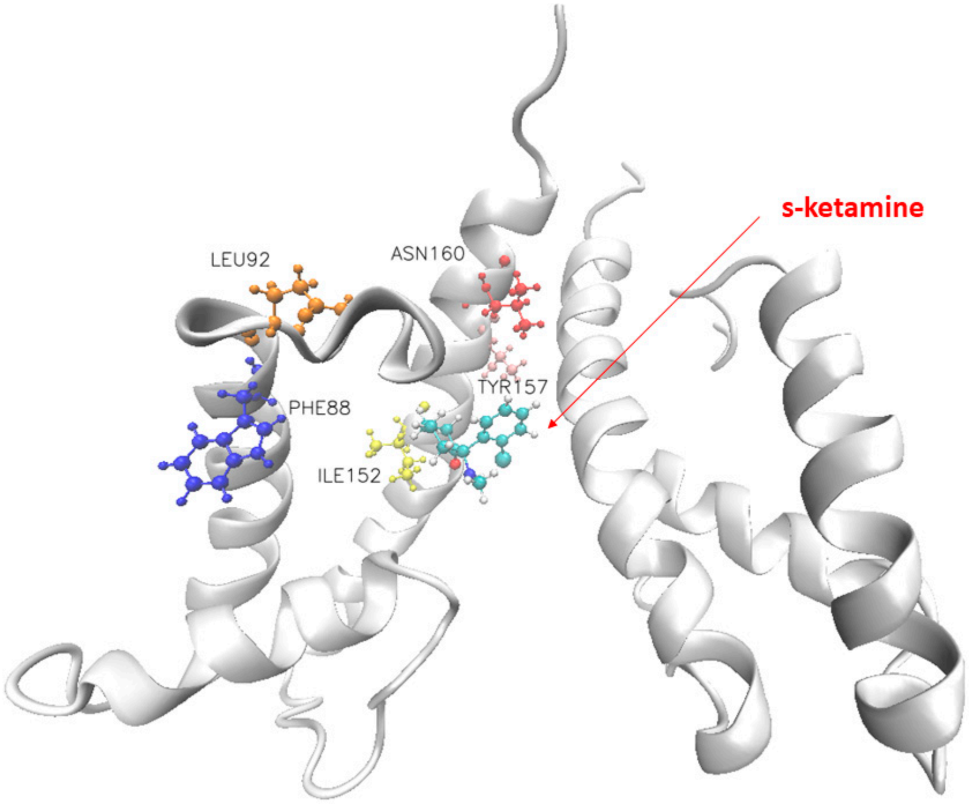

Figure S2 Visualization of the docking conformation of the NMDA receptor and s-ketamine. The ball-and-stick model shows the binding pocket of five residues in the NMDA receptor with dominant binding contributions to ketamine.

Table S3 Binding energy and decomposition of top 10 residues with dominant binding contributions of the NMDA receptor to s-ketamine, including van der Waals energy (vdW), electrostatic energy (Ele), polar solvation energy (Polar) and non-polar solvation energy (Non-polar).

Residue	TOTAL	van der Waals	Electrostatic	Polar Solvation	Non-Polar Solv.
**Phe88**	-2.19287	-2.04711	-1.12602	1.110792	-0.13053
**Asn160**	-2.14918	-0.94558	-4.3375	3.318178	-0.18428
**Leu92**	-1.78956	-1.93294	-0.47498	0.903851	-0.28549
**Tyr157**	-1.34733	-1.25785	-0.23522	0.212931	-0.06719
**Trp93**	-1.27813	-1.4686	-0.2309	0.501495	-0.08012
**Val96**	-0.99227	-0.93196	0.324644	-0.21474	-0.17021
**Ile153**	-0.8618	-0.76555	0.223871	-0.25334	-0.06678
**Ser156**	-0.58111	-1.14993	0.636683	0.152842	-0.2207
**Trp121**	-0.56147	-0.70984	0.034218	0.184792	-0.07064
**Gln89**	-0.44267	-0.92434	-0.57443	1.113663	-0.05757

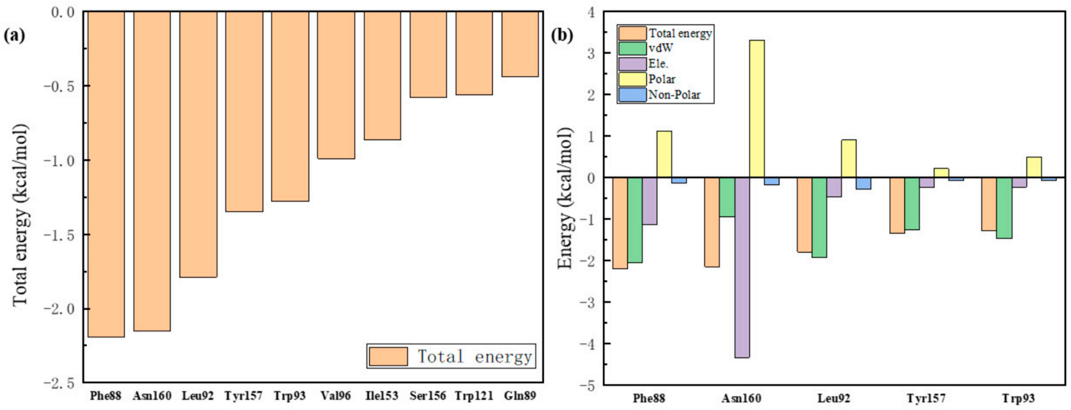

Figure S3 Decomposition of the free energy of binding of key residues. (a) The 10 key residues with dominant binding contributions from the NMDA receptor to s-ketamine. (b) Decomposition of the energy of the five key residues and s-ketamine pairs into four energy terms, namely van der Waals interaction (vdW), electrostatic interaction (ele), polar solvation energy (polar), and nonpolar energy (nonpolar).

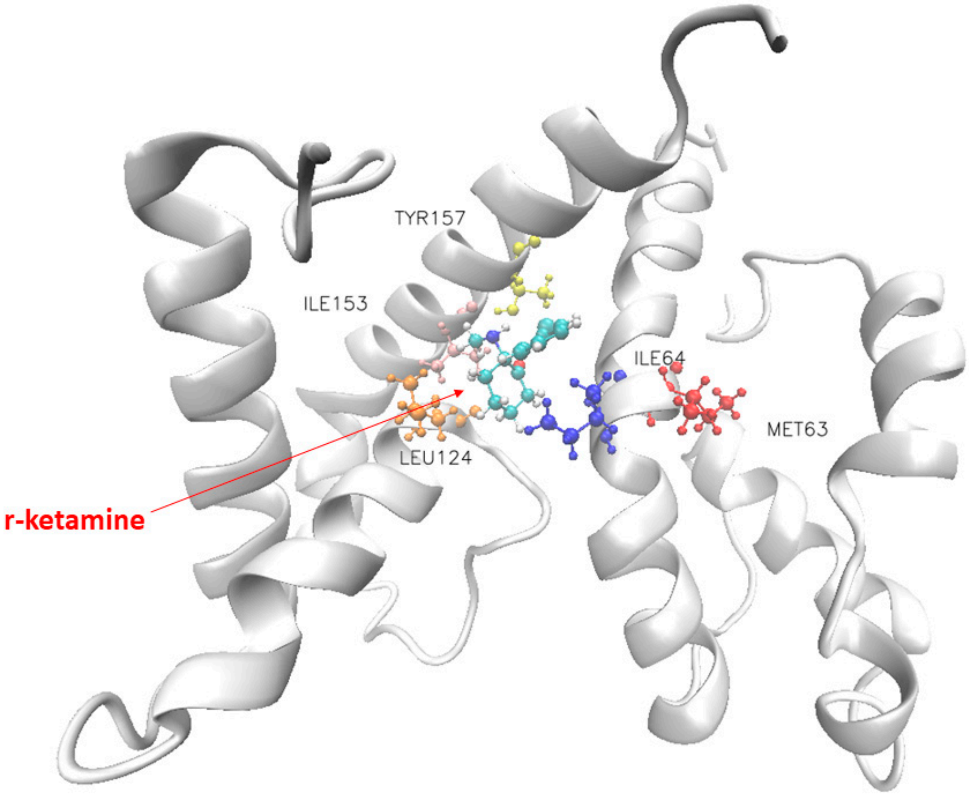

Fig S4 Visualization of the docking conformation of the NMDA receptor and r-ketamine. The ball-and-stick model shows the binding pocket of five residues in the NMDA receptor with dominant binding contributions to r-ketamine.

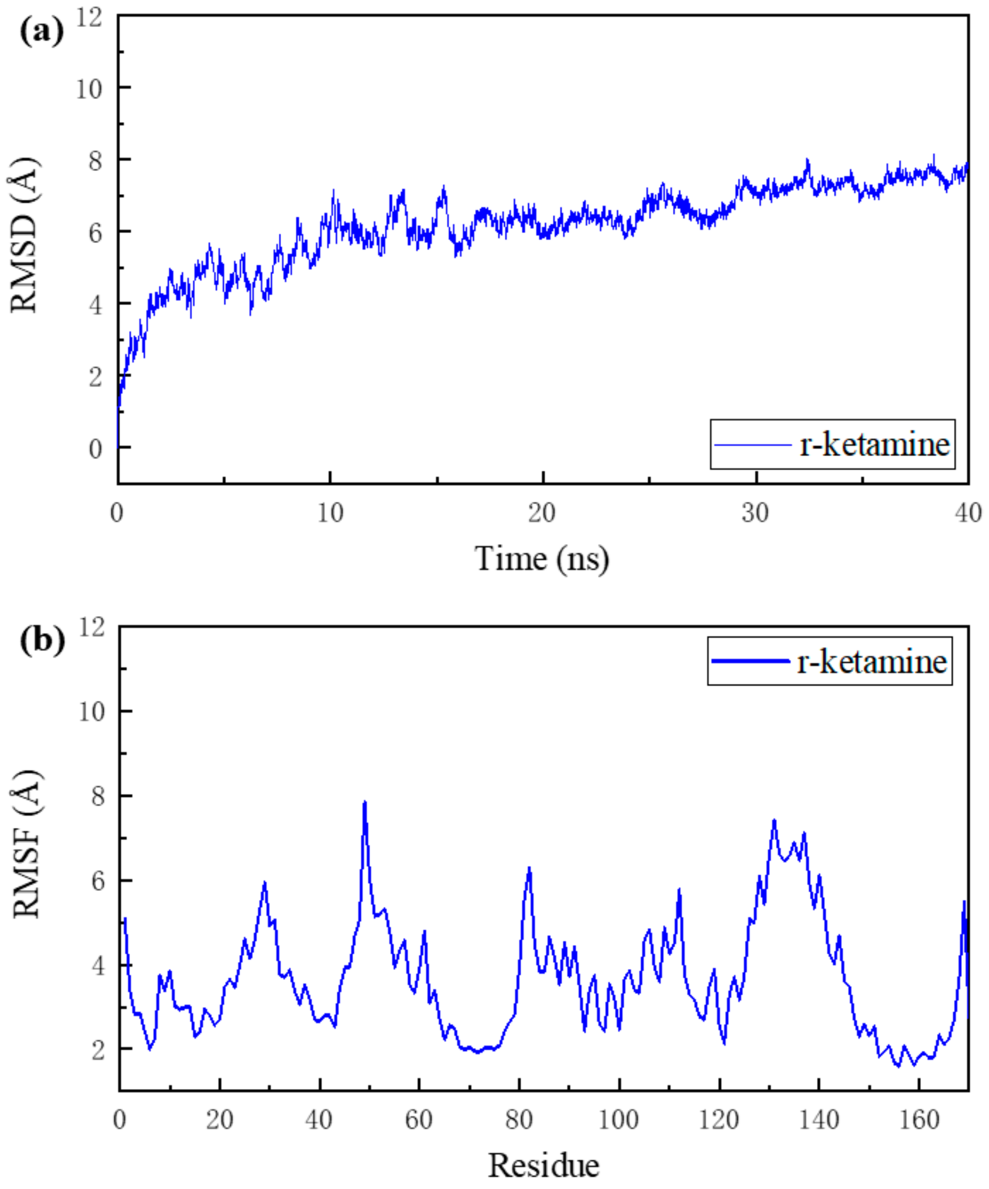

Figure S5 Root mean square deviation (RMSD) and root mean square fluctuation (RMSF) of r-ketamine and the NMDA receptor complex in a 40-ns simulation MD. (a) The RMSD of all non-hydrogen atoms in the complex. (b) The RMSF of all non-hydrogen atoms in the NMDA receptor throughout the simulation.

Table S4 Binding energy and decomposition of top 10 residues with dominant binding contributions of the NMDA receptor to r-ketamine, including van der Waals energy (vdW), electrostatic energy (Ele), polar solvation energy (Polar) and non-polar solvation energy (Non-polar).

Residue	TOTAL	van der Waals	Electrostatic	Polar Solvation	Non-Polar Solv.
**Met63**	-1.76638	-1.28128	-1.22127	0.824329	-0.08815
**Ile64**	-1.60067	-1.51763	-0.64395	0.767993	-0.20708
**Leu124**	-1.37106	-1.48867	-0.72941	1.008282	-0.16127
**Ile153**	-1.24179	-1.20975	-0.01193	0.119086	-0.13919
**Tyr157**	-1.11015	-1.32248	-0.07776	0.417628	-0.12753
**Val154**	-0.95168	-0.93403	-0.12226	0.206771	-0.10216
**Trp 146**	-0.68055	-0.96497	-0.23216	0.601987	-0.0854
**Leu125**	-0.65302	-0.72225	0.042748	0.106176	-0.0797
**Asn126**	-0.61274	-0.3845	-0.82881	0.668924	-0.06835
**Phe88**	-0.57836	-0.7225	0.032405	0.238734	-0.127

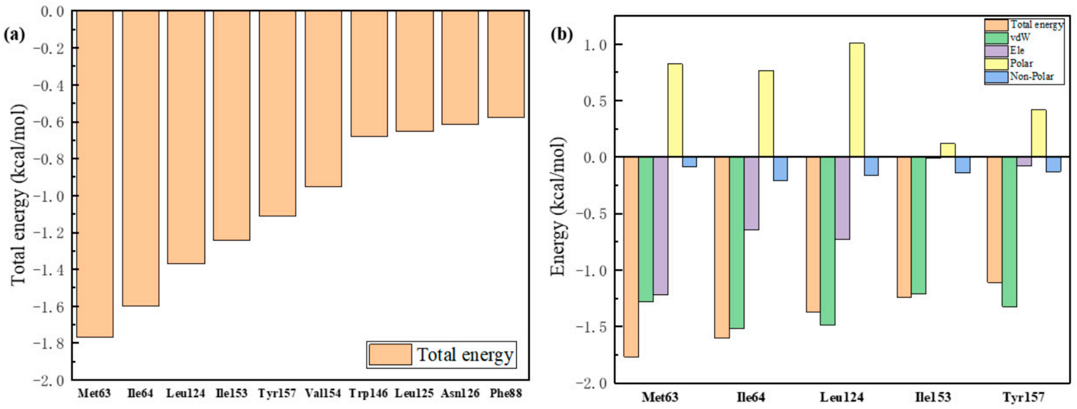

Figure S6 Decomposition of the free energy of binding of key residues. (a) The 10 key residues with dominant binding contributions from the NMDA receptor to r-ketamine. (b) Decomposition of the energy of the five key residues and r-ketamine pairs into four energy terms, namely van der Waals interaction (vdW), electrostatic interaction (ele), polar solvation energy (polar), and nonpolar energy (nonpolar).

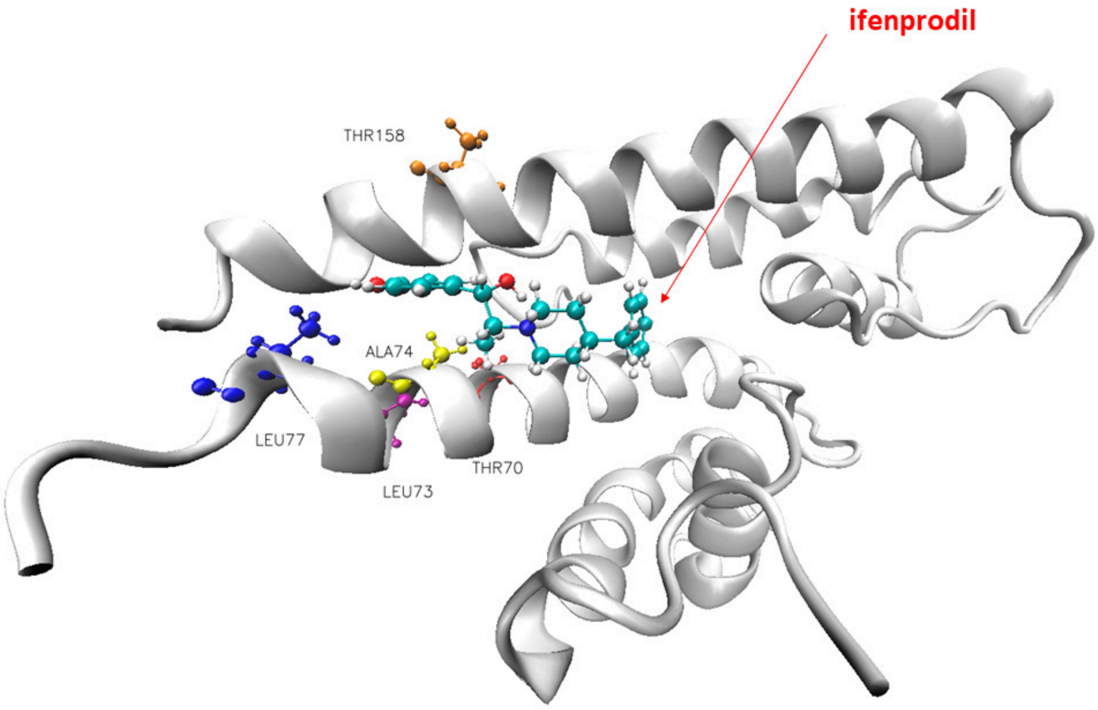

Figure S7 Visualization of the docking conformation of the NMDA receptor and ifenprodil. The ball-and-stick model shows the binding pocket of five residues in the NMDA receptor with dominant binding contributions to ifenprodil.

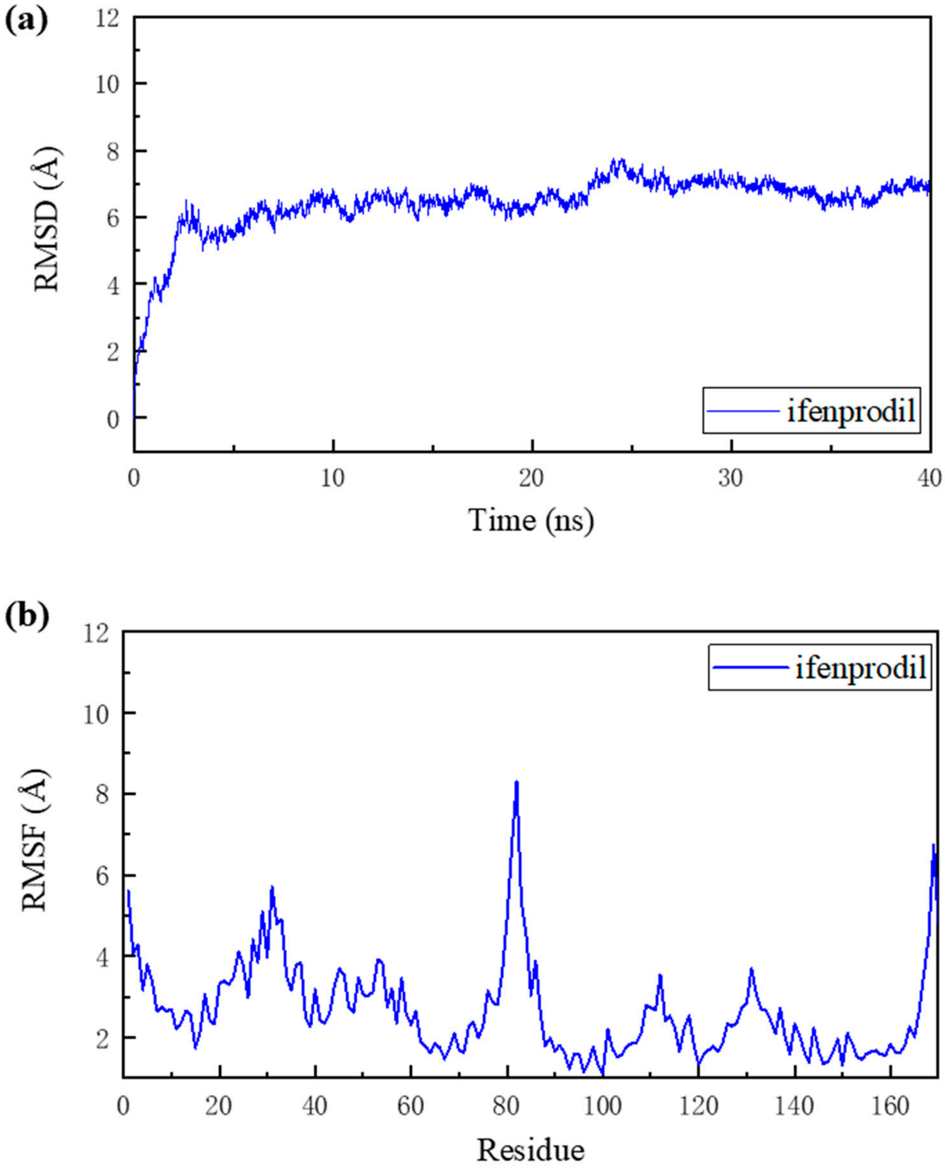

Figure S8 Root mean square deviation (RMSD) and root mean square fluctuation (RMSF) of ifenprodil and the NMDA receptor complex in a 40-ns simulation MD. (a) The RMSD of all non-hydrogen atoms in the complex. (b) The RMSF of all non-hydrogen atoms in the NMDA receptor throughout the simulation.

Table S5 Binding energy and decomposition of top 10 residues with dominant binding contributions of the NMDA receptor to ifenprodil, including van der Waals energy (vdW), electrostatic energy (Ele), polar solvation energy (Polar) and non-polar solvation energy (Non-polar).

Residue	TOTAL	van der Waals	Electrostatic	Polar Solvation	Non-Polar Solv.
**Leu77**	1.687922	-1.54449	-0.2605	0.427807	-0.31074
**Thr70**	1.583291	-1.2928	-0.07637	-0.06165	-0.15246
**Thr158**	1.410215	-1.59881	-0.00882	0.402392	-0.20498
**Leu 73**	1.375455	-1.14104	0.062973	-0.14098	-0.15641
**Ala74**	0.991262	-1.04664	-0.01878	0.163475	-0.08931
**Ala 162**	0.933872	-0.83479	-0.24005	0.309635	-0.16867
**Val 154**	0.717936	-0.64291	0.175276	-0.17372	-0.07658
**Leu 165**	0.684936	-0.39705	-0.56417	0.302684	-0.0264
**Leu 161**	0.64939	-0.49321	-0.06996	-0.06838	-0.01785
**Met 151**	0.635817	-0.63292	-0.11679	0.241465	-0.12757

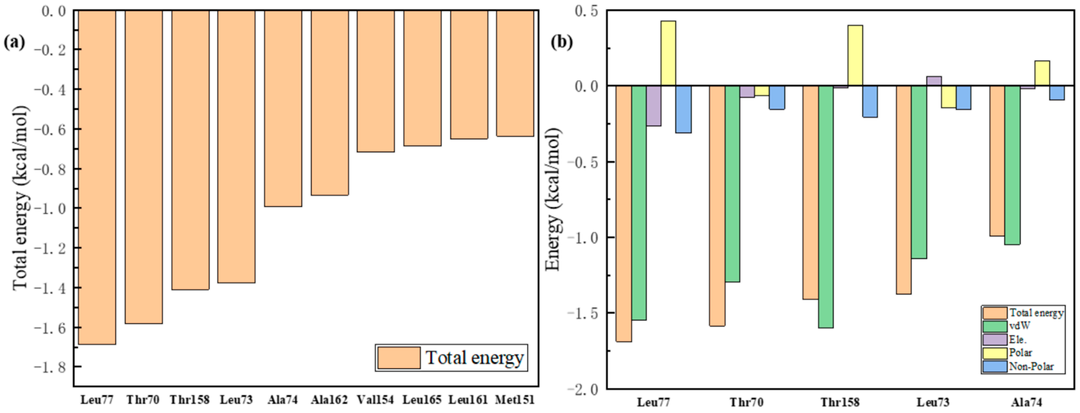

Figure S9 Decomposition of the free energy of binding of key residues. (a) The 10 key residues with dominant binding contributions from the NMDA receptor to ifenprodil. (b) Decomposition of the energy of the five key residues and ifenprodil pairs into four energy terms, namely van der Waals interaction (vdW), electrostatic interaction (ele), polar solvation energy (polar), and nonpolar energy (nonpolar).

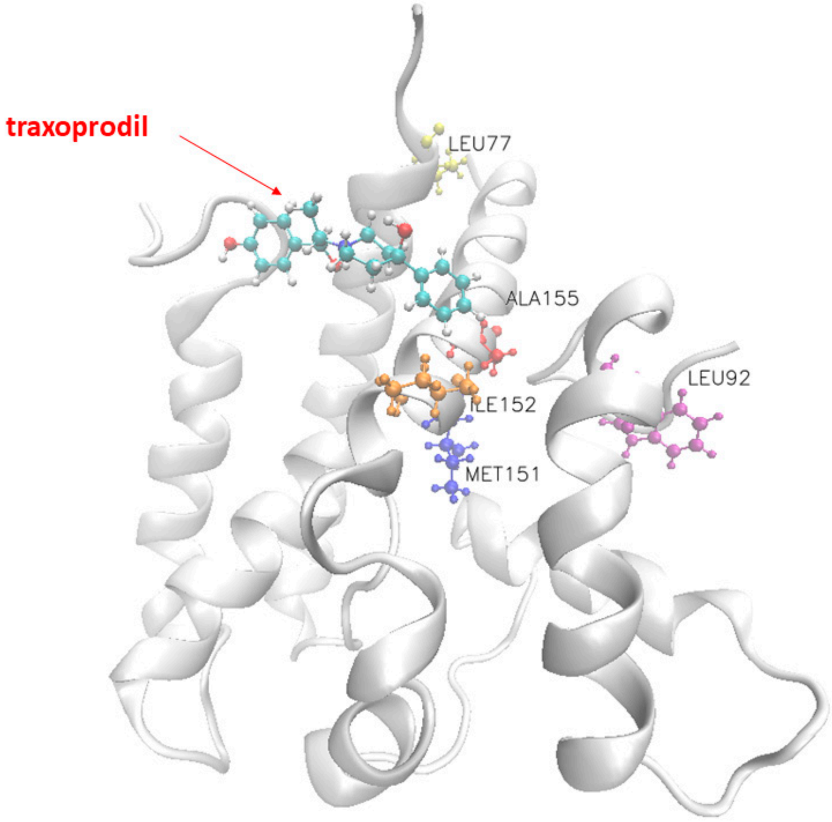

Figure S10 Visualization of the docking conformation of the NMDA receptor and traxprodil. The ball-and-stick model shows the binding pocket of five residues in the NMDA receptor with dominant binding contributions to traxprodil.

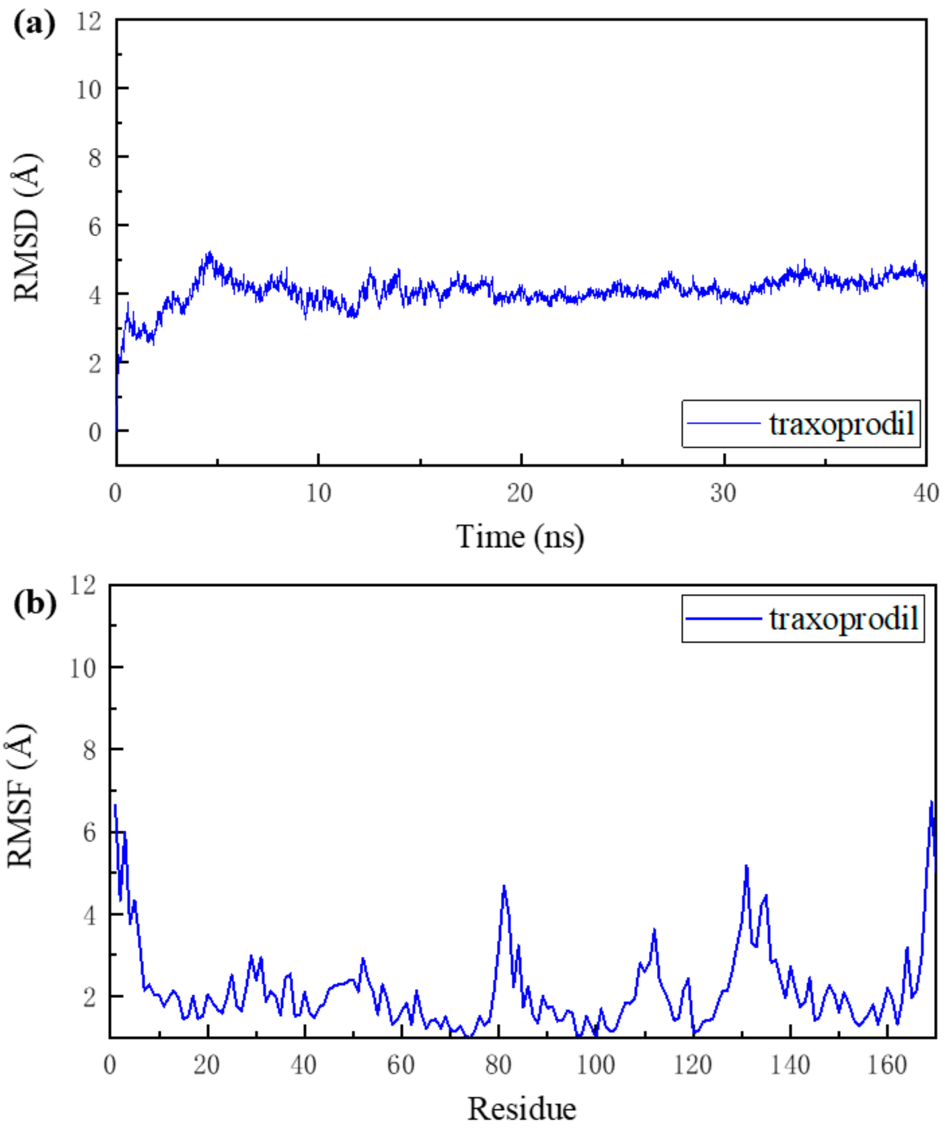

Figure S11 Root mean square deviation (RMSD) and root mean square fluctuation (RMSF) of traxprodil and the NMDA receptor complex in a 40-ns simulation MD. (a) The RMSD of all non-hydrogen atoms in the complex. (b) The RMSF of all non-hydrogen atoms in the NMDA receptor throughout the simulation.

Table S6 Binding energy and decomposition of top 10 residues with dominant binding contributions of the NMDA receptor to traxprodil, including van der Waals energy (vdW), electrostatic energy (Ele), polar solvation energy (Polar) and non-polar solvation energy (Non-polar).

Residue	TOTAL	van der Waals	Electrostatic	Polar Solvation	Non-Polar Solv.
**IleLE 152**	1.574005	-1.47964	0.002555	0.161645	-0.25856
**Ala 155**	1.50332	-1.23123	-0.23519	0.128362	-0.16526
**Leu77**	1.178284	-1.27187	-0.18614	0.51792	-0.23819
**Leu92**	0.85404	-0.7672	-0.01114	0.096595	-0.17229
**Met 151**	0.826756	-0.8536	-0.00697	0.186957	-0.15314
**Met4**	0.632208	-0.50579	-0.28928	0.237581	-0.07472
**Pro6**	0.607193	-0.59218	-0.30466	0.362017	-0.07237
**Ala 159**	0.572672	-0.54884	-0.03852	0.099731	-0.08504
**Phe76**	0.528461	-0.63222	-0.0879	0.311136	-0.11948
**Leu73**	0.515525	-0.46042	-0.04371	0.037983	-0.04938

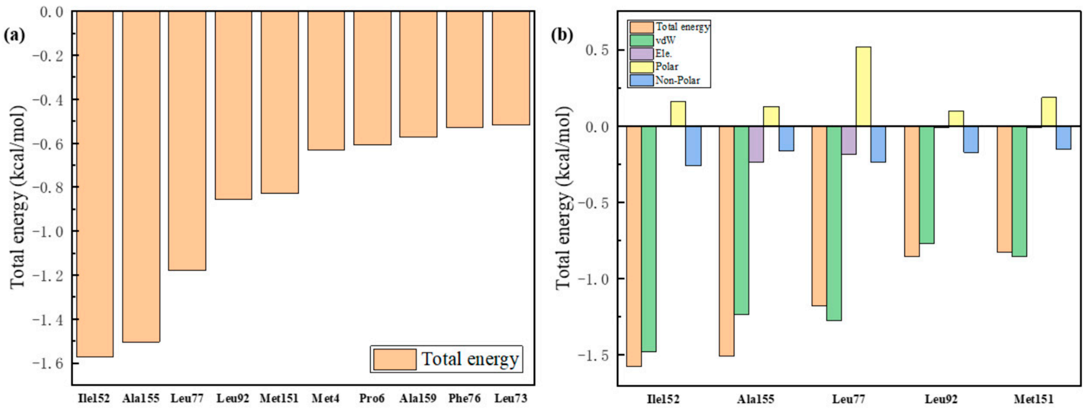

Figure S12 Decomposition of the free energy of binding of key residues. (a) The 10 key residues with dominant binding contributions from the NMDA receptor to traxprodil. (b) Decomposition of the energy of the five key residues and traxprodil pairs into four energy terms, namely van der Waals interaction (vdW), electrostatic interaction (ele), polar solvation energy (polar), and nonpolar energy (nonpolar).

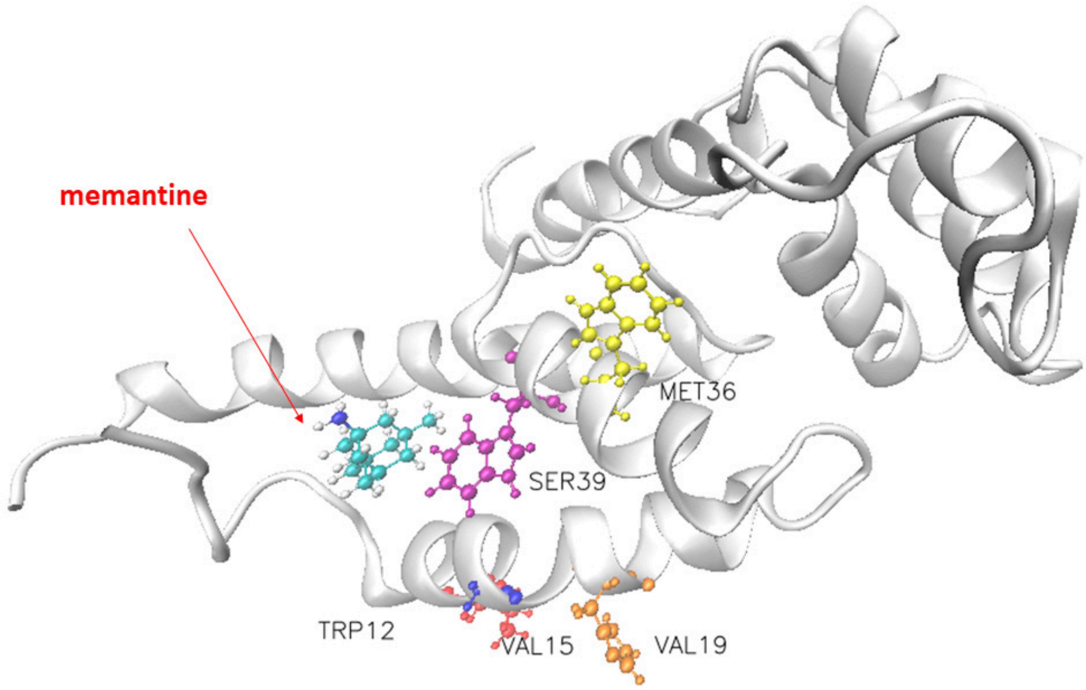

Figure S13 Visualization of the docking conformation of the NMDA receptor and memantine. The ball-and-stick model shows the binding pocket of five residues in the NMDA receptor with dominant binding contributions to memantine.

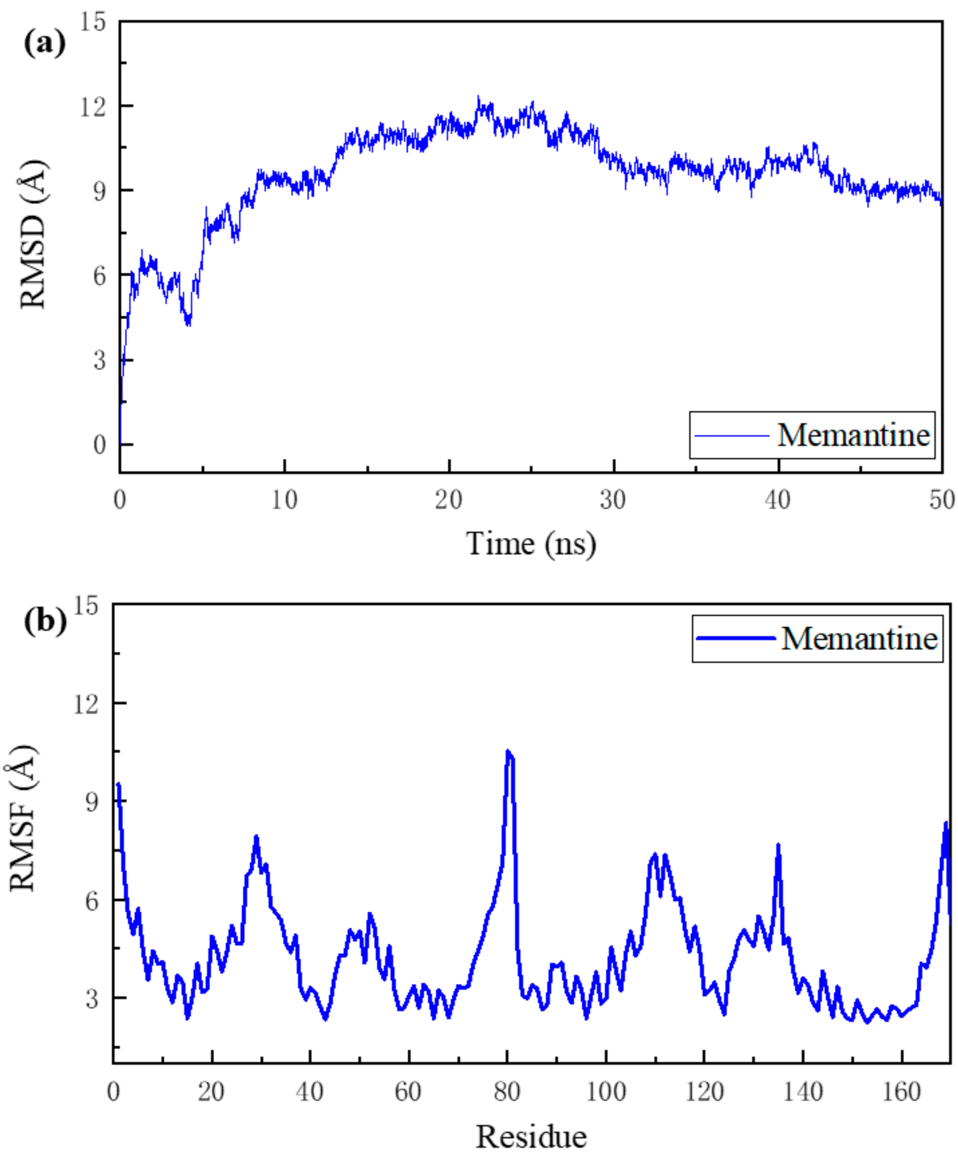

Figure S14 Root mean square deviation (RMSD) and root mean square fluctuation (RMSF) of memantine and the NMDA receptor complex in a 50-ns simulation MD. (a) The RMSD of all non-hydrogen atoms in the complex. (b) The RMSF of all non-hydrogen atoms in the NMDA receptor throughout the simulation.

Table S7 Binding energy and decomposition of top 10 residues with dominant binding contributions of the NMDA receptor to memantine, including van der Waals energy (vdW), electrostatic energy (Ele), polar solvation energy (Polar) and non-polar solvation energy (Non-polar)

Residue	TOTAL	van der Waals	Electrostatic	Polar Solvation	Non-Polar Solv.
**Met36**	-1.22973	-1.32584	-0.12447	0.452622	-0.23204
**Val15**	-1.1356	-0.98899	0.04405	-0.12713	-0.06353
**Trp12**	-0.95294	-1.6389	-0.16025	1.02093	-0.17472
**Val19**	-0.93217	-0.7985	0.014264	-0.05241	-0.09551
**Trp40**	-0.91198	-1.61507	-0.19885	1.071532	-0.16959
**Gly16**	-0.67071	-0.69202	-0.00529	0.159731	-0.13312
**Met4**	-0.64472	-0.71532	-0.04542	0.267005	-0.15098
**Ser39**	-0.42161	-0.50578	0.012915	0.114542	-0.04329
**Leu43**	-0.31954	-0.30826	0.022955	-0.00696	-0.02728
**Leu13**	-0.23787	-0.2985	-0.01988	0.110294	-0.02979

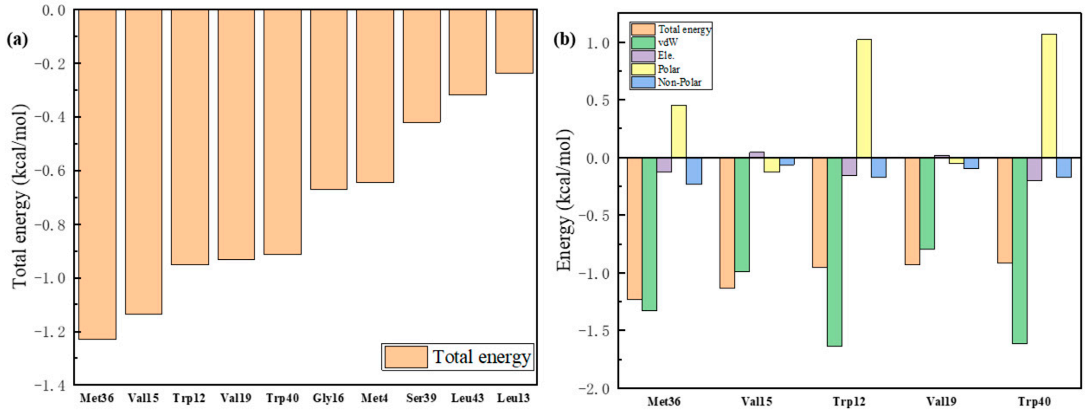

Figure S15 Decomposition of the free energy of binding of key residues. (a) The 10 key residues with dominant binding contributions from the NMDA receptor to memantine. (b) Decomposition of the energy of the five key residues and memantine pairs into four energy terms, namely van der Waals interaction (vdW), electrostatic interaction (ele), polar solvation energy (polar), and nonpolar energy (nonpolar).

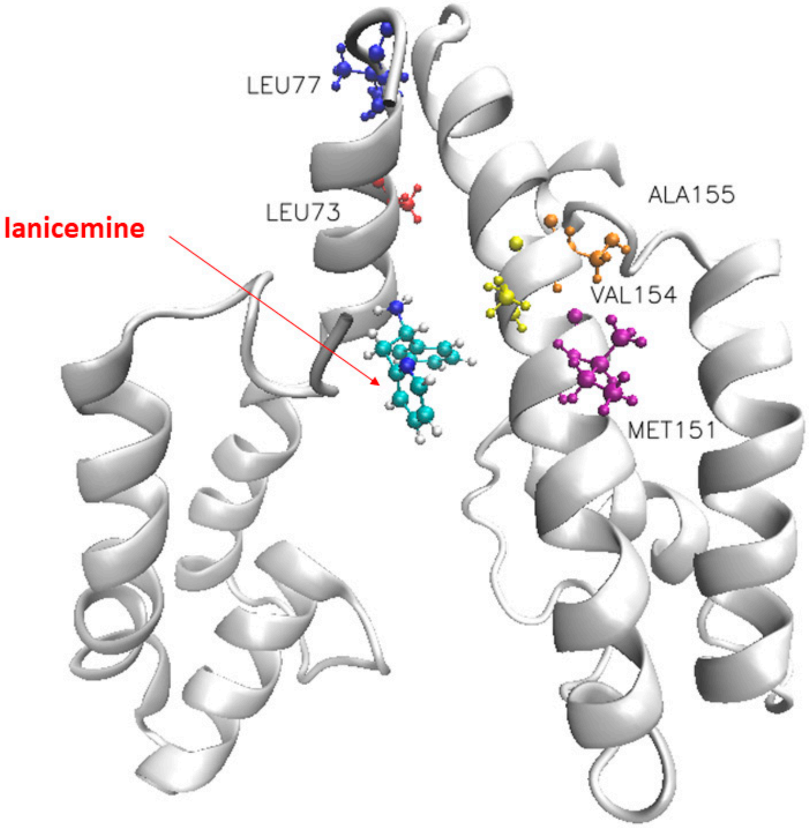

Figure S16 Visualization of the docking conformation of the NMDA receptor and lanicemine. The ball-and-stick model shows the binding pocket of five residues in the NMDA receptor with dominant binding contributions to lanicemine.

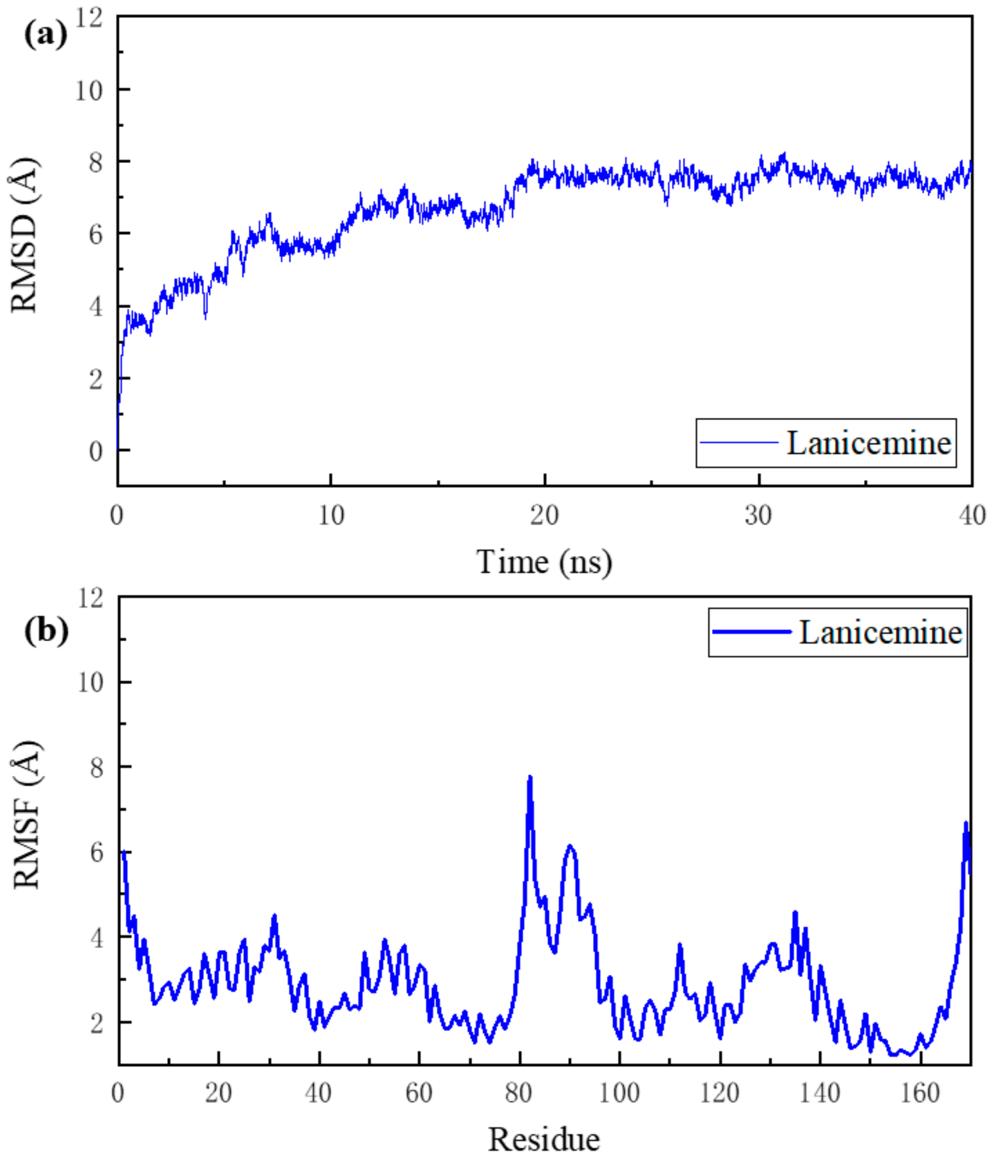

Figure S17 Root mean square deviation (RMSD) and root mean square fluctuation (RMSF) of lanicemine and the NMDA receptor complex in a 40-ns simulation MD. (a) The RMSD of all non-hydrogen atoms in the complex. (b) The RMSF of all non-hydrogen atoms in the NMDA receptor throughout the simulation.

Table S8 Binding energy and decomposition of top 10 residues with dominant binding contributions of the NMDA receptor to lanicemine, including van der Waals energy (vdW), electrostatic energy (Ele), polar solvation energy (Polar) and non-polar solvation energy (Non-polar).

Residue	Total energy	van der Waals	Electrostatic	Polar Solvation	Non-Polar Solv.
**Ala155**	-1.38505	-1.26383	-0.24229	0.345445	-0.22439
**Leu73**	-0.74935	-0.66406	0.055282	-0.01769	-0.12289
**Met151**	-0.72665	-0.75208	-0.01362	0.191402	-0.15235
**Leu77**	-0.70765	-0.67469	-0.00131	0.137066	-0.16871
**Val154**	-0.67319	-0.57298	-0.04368	0.009777	-0.0663
**Thr158**	-0.45623	-0.5595	-0.13422	0.267106	-0.02961
**Ile 152**	-0.44839	-0.39728	-0.03973	0.052193	-0.06358
**Ala159**	-0.39072	-0.37716	-0.01477	0.083784	-0.08258
**Phe3**	-0.38615	-0.46228	-0.09865	0.280917	-0.10614
**Thr70**	-0.19303	-0.28424	-0.07625	0.191146	-0.02369

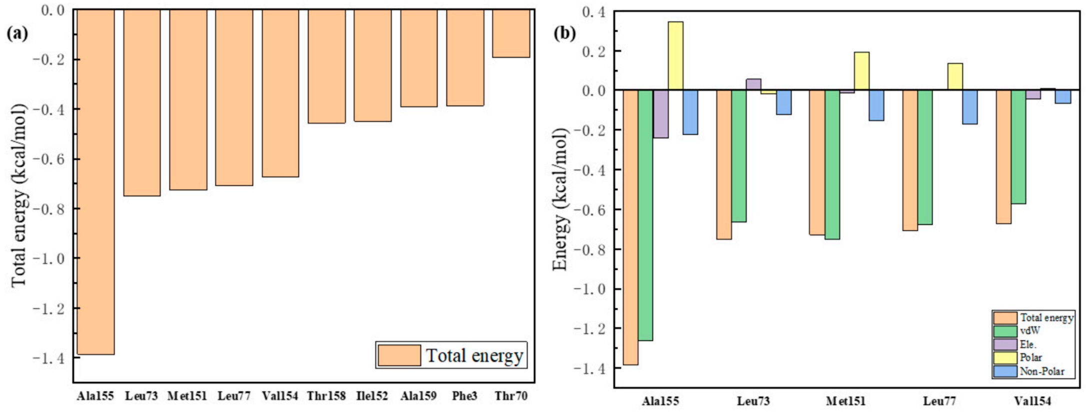

Figure S18 Decomposition of the free energy of binding of key residues. (a) The 10 key residues with dominant binding contributions from the NMDA receptors to lanicemine. (b) Decomposition of the energy of the five key residues and lanicemine pairs into four energy terms, namely van der Waals interaction (vdW), electrostatic interaction (ele), polar solvation energy (polar), and nonpolar energy (nonpolar).

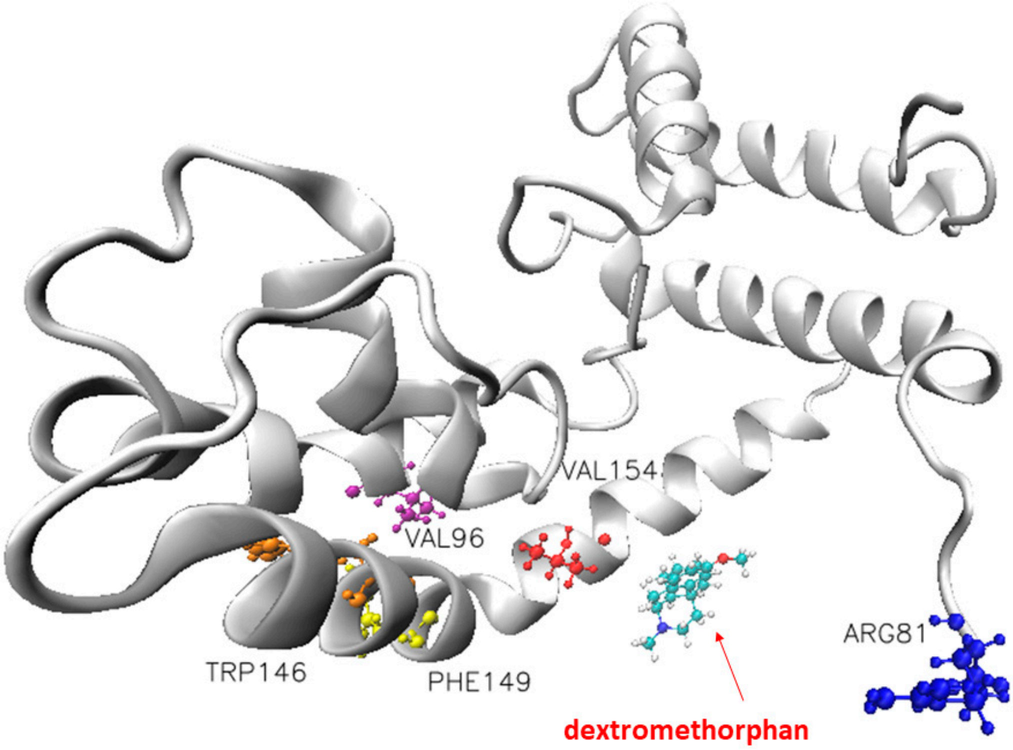

Figure S19 Visualization of the docking conformation of the NMDA receptor and dextromethorphan. The ball-and-stick model shows the binding pocket of five residues in the NMDA receptor with dominant binding contributions to dextromethorphan.

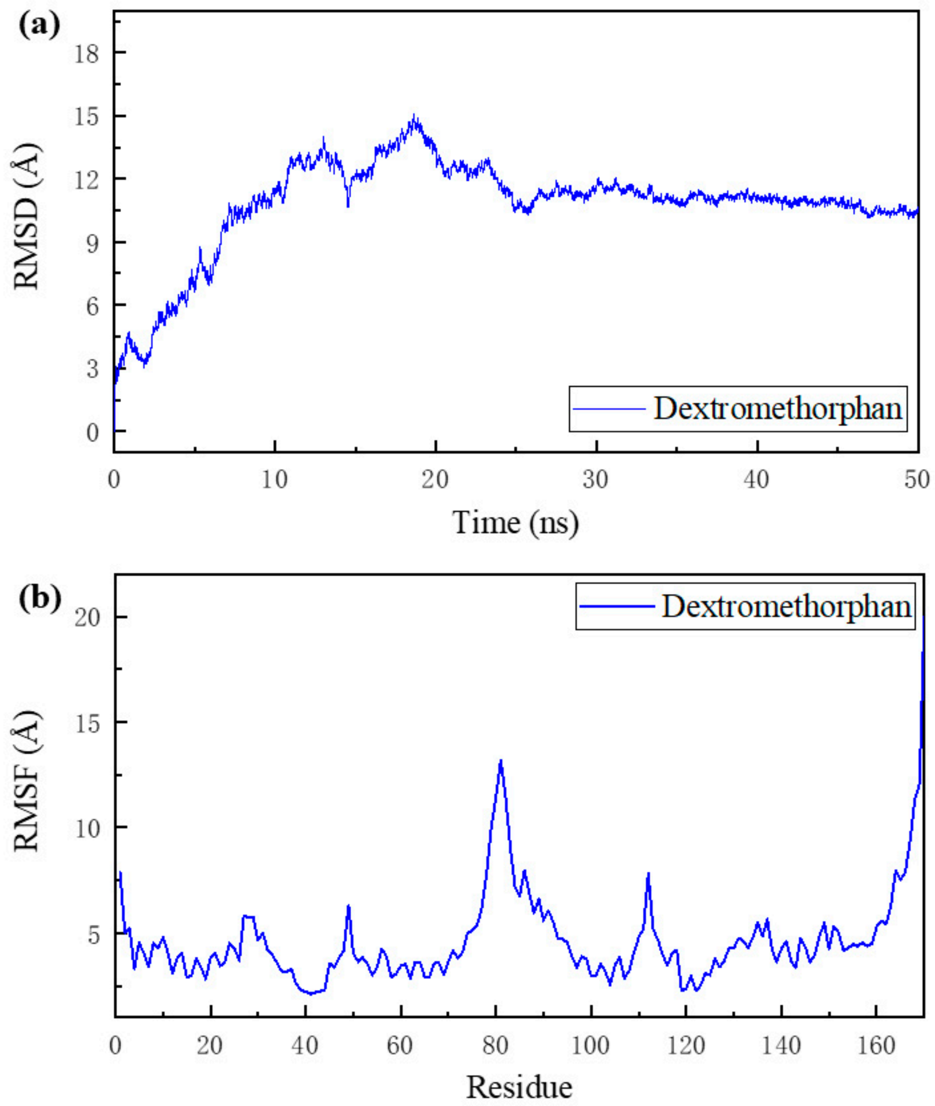

Figure S20 Root mean square deviation (RMSD) and root mean square fluctuation (RMSF) of dextromethorphan and the NMDA receptor complex in a 50-ns simulation MD. (a) The RMSD of all non-hydrogen atoms in the complex. (b) The RMSF of all non-hydrogen atoms in the NMDA receptor throughout the simulation.

Table S9 Binding energy and decomposition of top 10 residues with dominant binding contributions of NMDA receptor to dextromethorphan, including van der Waals energy (vdW), electrostatic energy (Ele), polar solvation energy (Polar) and non-polar solvation energy (Non-polar).

Residue	TOTAL	van der Waals	Electrostatic	Polar Solvation	Non-Polar Solv.
**Val96**	-0.5848	-0.51287	-0.01389	0.03004	-0.08808
**Phe149**	-0.54639	-0.74609	0.029353	0.311368	-0.14101
**Trp146**	-0.34524	-0.61197	-0.03936	0.387806	-0.08171
**Arg81**	-0.34509	-0.57003	-0.23569	0.556045	-0.09541
**Val154**	-0.26438	-0.24528	-0.02877	0.041169	-0.0315
**Ser99**	-0.25508	-0.31555	-0.16573	0.298333	-0.07213
**Val100**	-0.20423	-0.20154	-0.00052	0.012124	-0.0143
**Ile153**	-0.17272	-0.14407	-0.02008	0.001572	-0.01013
**Val103**	-0.15839	-0.13281	-0.00159	-0.00228	-0.02172
**Leu77**	-0.15532	-0.15283	0.003647	0.022507	-0.02864

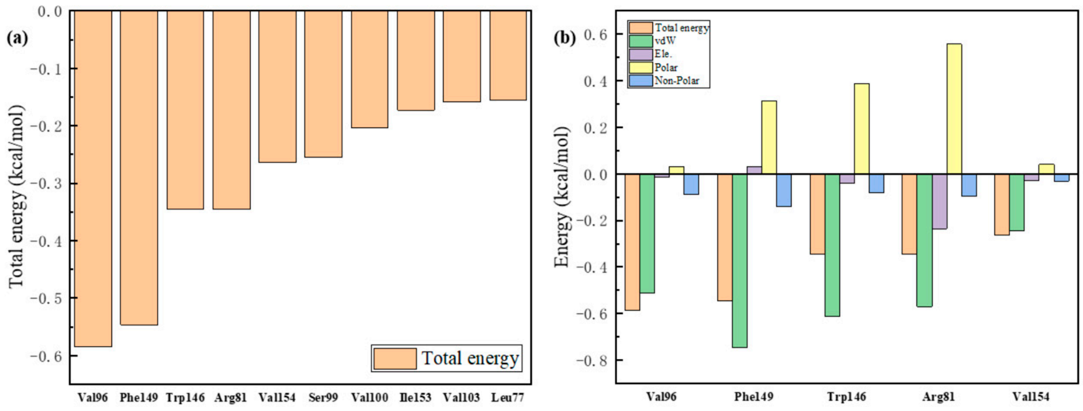

Figure S21 Decomposition of the free energy of binding of key residues. (a) The 10 key residues with dominant binding contributions from the NMDA receptor to dextromethorphan. (b) Decomposition of the energy of the five key residues and dextromethorphan pairs into four energy terms, namely van der Waals interaction (vdW), electrostatic interaction (ele), polar solvation energy (polar), and nonpolar energy (nonpolar).
